# Clinical case of polymicrobial and multidrug-resistant posttraumatic chronic hip infection treatment

**DOI:** 10.5194/jbji-11-565-2026

**Published:** 2026-09-10

**Authors:** Urban Vadnjal, Samo Roškar, René Mihalič, Mitja Rak, Rihard Trebše

**Affiliations:** 1 Valdoltra Orthopaedic Hospital, Jadranska cesta 31, 6280 Ankaran, Slovenia; 2 Faculty of Medicine, University of Ljubljana, Korytkova ulica 2, 1000 Ljubljana, Slovenia; 3 Centre for Medical Microbiology, National Laboratory of Health, Environment and Food, Verdijeva 11, 6000 Koper, Slovenia

## Abstract

A 40-year-old man, after a left acetabular fracture, had eight surgeries, suffered from chronic multidrug-resistant infection with fistula, and underwent multi-stage hip revision surgery with spacer exchange and reconstruction, combined with systemic and intra-articular antibiotic therapy. He remained infection-free at 3.5-year follow-up.

## 1 Introduction

Musculoskeletal infections related to orthopaedic-related devices, either as a fracture-related infection (FRI) or periprosthetic joint infection (PJI), are associated with biofilm formation, which itself is considered to be a devastating complication associated with poor functional outcomes and high mortality rates (McNally et al., 2021; Moriarty et al., 2022). The incidence of PJI varies among different centres and the joint involved; for total hip arthroplasty (THA) it is reported to be between 0.5 % and 2 % for primary THA and up to 15 % for revision THA (Mian et al., 2022). Particularly in patients with PJI, it is crucial to avoid dead-end clinical situations such as amputations, which could result in a catastrophic functional outcome (Hantouly et al., 2024). In PJI the most critical factor influencing the success of PJI treatment is biofilm eradication, which acts as physical barrier against antibiotics penetrating into the site of action, and, thus, for many bacterial species, minimal biofilm eradication concentration is up to 1000 times higher than the minimal inhibitory concentration (Macià et al., 2014). An additional emerging problem among PJI and FRI is multidrug resistance (MDR) of the causative pathogens (Siljander et al., 2018). Of particular concern are carbapenem-resistant Enterobacterales (CRE), including carbapenemase-producing Enterobacterales (CPE), as well as carbapenem-resistant *Acinetobacter baumannii* (CRAb), which may also produce carbapenemases (CP) (Paul et al., 2022). In the setting of MDR infection, particularly in the presence of biofilm, treatment of PJI remains a major clinical challenge. Reports on MDR bacteria causing PJI are rare. In recent years, two possible solutions for the treatment of MDR PJI were provided in the literature, one with the use of phage therapy and another with the use of local antibiotics (Vandeputte et al., 2025). The use of intra-articular antibiotics was first described by Perry and Pearson in 1992 and popularise by Whiteside and Roy in 2017 as an approach toward antibiotic delivery to avoid systemic toxicity, poor local perfusion, and insufficient immune response around the infected joint (Perry et al., 1992; Whiteside and Roy, 2017). This article reports a rare clinical case of PJI of hip joint infection with MDR bacteria that was successfully treated with a multistage revision protocol combined with a combination of systemic and local antibiotics.

## 2 Case history

**Figure 1:**
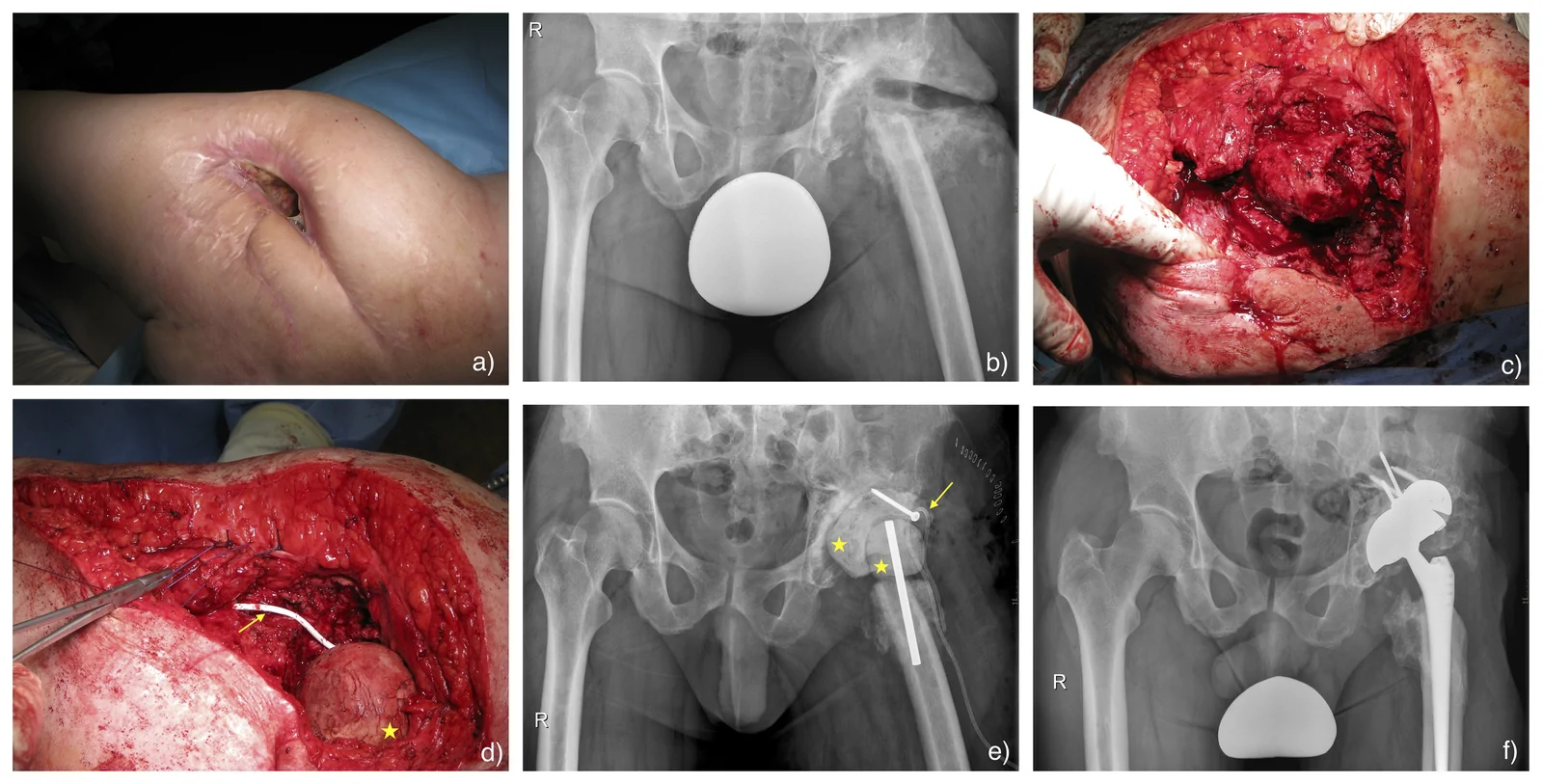
**(a)** Extensive cutaneous sinus tract on the lateral aspect of the left hip. **(b)** Radiograph at referral demonstrating heterotopic ossification and sinus tract communication to the acetabulum, following resection of the proximal femur and greater trochanter. **(c)** Intraoperative view of the exposed infection focus prior to debridement. **(d)** Intraoperative view following debridement, with an antibiotic-impregnated cement spacer ( ⋆ ) and intra-articular catheter ( → ) in situ. **(e)** Postoperative radiograph following debridement, demonstrating two-part antibiotic cement spacer ( ⋆⋆ ) and intra-articular catheter ( → ) within the joint cavity. **(f)** Postoperative radiograph at most recent follow-up.

A 40-year-old healthy man was referred to our institution in 2021 with an extensive sinus tract following total hip arthroplasty explantation. He had sustained a left acetabular fracture as part of a polytrauma in 2021 and underwent treatment elsewhere for osteosynthesis, which failed, with the patient requiring eight additional surgeries due to FRI and PJI, resulting in chronic MDR infection. Detailed records from prior interventions were unavailable. Written informed consent was provided by the patient for this paper to be published. The major findings at admission were fist-size sinus tract on the lateral part of the left hip with 15 cm shortening (Fig. 1a). The imaging showed a sinus tract to the acetabulum with heterotrophic ossification and the state after resection of the proximal femur together with the greater trochanter (Fig. 1b). C-reactive protein (CRP) was 12.8 mg L^−1^. According to the institutional algorithm (Trebše and Roškar, 2021), debridement with an antibiotic (0.5 g gentamicin) spacer in the left hip was performed (Fig. 1c, d, and e). Four sachets of gentamicin- and clindamycin-loaded bone cement (42.9 g each) were used, supplemented with the addition of vancomycin 4 g and meropenem 2 g.

Intraoperatively, nine samples for microbiological and three samples for histological analysis were obtained. After tissue sampling, empirical antibiotic therapy was initiated with intravenous piperacillin-tazobactam 4.5 g q8h and vancomycin (starting dose of 2 g, with the dosage being adjusted according to plasma concentration levels). Microbiological analysis of intraoperative biopsies identified several MDR bacteria, OXA-48-producing carbapenem-resistant *Klebsiella pneumoniae* (positive in two out of nine microbiological samples) and carbapenem-resistant, carbapenemase-producing *Acinetobacter baumannii* (eight out of nine), alongside *Corynebacterium* *amycolatum* (one out of nine), *Corynebacterium striatum *(two out of nine), and * Staphylococcus epidermidis* (one out of nine), all of them resistant to methicillin and *Enterococcus faecalis* (two out of nine). According to the antibiograms of isolated bacteria, on the third day after surgery, antibiotic treatment was upscaled tovancomycin, fosfomycin 5 g q8h, and colistin, with a loading dose of 9 MIU, followed by a maintenance dose of 4.5 MIU q12h, as well as intra-articular vancomycin at 400 mg per 5 mL q24h and colistin at 125 000 IU per 2 mL q24h was commenced. Intra-articular vancomycin and colistin doses were prepared and established by the pharmacy department based on physicochemical properties, cytotoxicity profiles, and drainage fluid sample concentrations with the aim of achieving the smallest possible volume in which the antibiotic remained fully soluble. Fosfomycin was discontinued 1 week postoperatively upon microbiological confirmation of resistance at 50 mg per 12 h of intravenous tigecycline with a loading dose of 100 mg, followed by the initiation of a maintenance dose of 50 mg q12h; 2 weeks postoperatively, intravenous and intra-articular vancomycin, as well as tigecycline, were discontinued, and intravenous teicoplanin 400 mg q24h and ceftazidime/avibactam 2 g per 0.5 g q8h were initiated. Upon receipt of the final microbiological results with definitive resistance profiles at 18 d postoperatively, therapy was continued with intravenous ceftazidime/avibactam 2 g per 0.5 g q8h (targeting *Klebsiella pneumoniae)* and vancomycin (targeting *Corynebacterium* *amycolatum*, *Corynebacterium striatum, Staphylococcus epidermidis*, and *Enterococcus faecalis*), dose-adjusted according to plasma concentration monitoring, combined with intra-articular colistin at 125 000 IU per 2 mL q24h (targeting *Acinetobacter baumannii*), representing the regimen with coverage against all isolated pathogens; 3 weeks after surgery, all intravenous and intra-articular antibiotic therapy was stopped, and, at that time, CRP was < 5 mg L^−1^. Intra-articular catheter management followed standard drainage tube protocols with regular dressing changes, with a watertight seal throughout the 3-week intra-articular antibiotic treatment period, after which the catheter was removed. In January 2022, after 2 weeks of antibiotic holiday, additional revision with debridement, tissue sampling, and spacer exchange was performed. Three sachets of gentamicin- and clindamycin-loaded bone cement (42.9 g each) were used, supplemented with the addition of vancomycin 3 g and colistin 3 MIU. Intravenous vancomycin (dose-adjusted according to plasma concentration monitoring) and ceftazidime/avibactam at 2 g per 0.5 g q8h IV, combined with intra-articular colistin at 125 000 IU per 2 mL q24h, were administered for 7 d. The samples were sterile, and there was no histological evidence of acute inflammation. At the end of February 2022, we performed the reconstruction of the hip with trabecular metal augments and dual-mobility typing of the shell and a cement-less femoral component (Fig. 1f). Intravenous vancomycin (dose-adjusted according to plasma concentration monitoring) and ceftazidime/avibactam at 2 g per 0.5 g q8h IV were administered for 5 d. As no susceptible oral antibiotic options were available, the entire treatment course was administered parenterally, with no oral antibiotics being used at any point. Intraoperative samples were, again, sterile. Serum creatinine, urea, and electrolytes were monitored regularly throughout the treatment course. At discharge, the patient was capable of walking with crutches, his surgical wound having healed. At the most recent follow-up in June 2026, no clinical or laboratory signs of infection were present. The patient ambulated without assistive devices for shorter distances, with good hip mobility and strength and absent resting pain with mild exertional discomfort. The Harris hip score was 78. The primary functional limitation was a foot drop and Achilles tendon contracture secondary to the index polytrauma. Radiographic evaluation demonstrated stable implant position with no osteolysis, loosening, or periprosthetic fracture. Limb length discrepancy was estimated to be approximately 2 cm.

## 3 Discussion

Musculoskeletal infections, when related to some type of the implant in the human body, either as a fracture-related infection (FRI) or as a periprosthetic joint infection (PJI), are considered to be a devastating complication in modern orthopaedic surgery (McNally et al., 2021; Moriarty et al., 2022). Particularly in patients with PJI, it is crucial to avoid dead-end clinical situations such as amputations, which could result in a catastrophic functional outcome (Hantouly et al., 2024). In PJI the most critical factor influencing the success of PJI treatment is biofilm eradication, which acts as physical barrier against antibiotics penetrating into the site of action, and, thus, for many bacterial species, minimal biofilm eradication concentration is up to 1000 times higher than the minimal inhibitory concentration (Macià et al., 2014). When dealing with Gram-negative bacteria, carbapenems are considered to be the last-line treatment option (Paul et al., 2022). Therefore, Gram-negative pathogens such as CRE CPE (OXA-48–producing *Klebsiella pneumoniae*) and CRAb-CP (*Acinetobacter baumannii*) have very limited therapeutic options. In such situations, the use of intra-articular antibiotics as described by Perry and Pearson in 1991 and popularise by Whiteside and Roy in 2017 should be considered to be a salvage option for biofilm treatment in addition to meticulous surgical debridement of the involved joint (Perry et al., 1992; Whiteside and Roy, 2017). The use of local delivery of antibiotics allows one to avoid systemic toxicity, poor local perfusion, and insufficient immune response around the infected joint (Aparicio-Blanco et al., 2024). Our case suggests that staged revision surgery combined with targeted systemic and local antibiotic therapy may represent a viable treatment approach for biofilm-associated PJI caused by MDR bacteria. However, the successful outcome was likely to be multifactorial, with important contributing factors including extensive debridement, multiple revision procedures, spacer exchange, systemic combination antimicrobial therapy, and careful multidisciplinary management. Therefore, the role of intra-articular antibiotics alone cannot be independently assessed from this single case.

## 4 Summary

The case demonstrates the importance of having well-established hospital algorithms for FRI and PJI treatment and strictly following them at each step. In most severe cases related to MDR bacteria, multiple-stage protocol is indicated, and intra-articular infusions of antibiotics should be considered in addition to standard intravenous regimens.

## Data Availability

No data sets were used in this article.
